# Hypoproteinemia and Hypogammaglobulinemia Due to a Homozygous *FCGRT* Mutation

**DOI:** 10.1007/s10875-026-02032-y

**Published:** 2026-06-08

**Authors:** Abdul Samet Ala, Bernice Lo, Ahmet Ozen, Ali Islek, Gokhan Tumgor

**Affiliations:** 1 Department of Pediatric Gastroenterology, Cukurova University School of Medicine, Adana, 01320 Turkey; 2 Division of Translational Medicine, Research Branch, Sidra Medicine, Doha, Qatar; 3 Department of Pediatric Allergy and Immunology, Marmara University School of Medicine, Istanbul, Turkey

To the Editor,

The neonatal Fc receptor (FcRn) plays an essential role in regulating immunoglobulin G (IgG) and albumin homeostasis. By rescuing these proteins from intracellular degradation and recycling them back into the circulation, FcRn prolongs their serum half-life. Disruption of this pathway leads to accelerated catabolism, resulting in reduced circulating IgG and albumin levels despite preserved synthesis [1].

FcRn is a heterodimer consisting of a heavy chain encoded by *FCGRT* and a light chain, β2-microglobulin, encoded by B2M. Human evidence for FcRn dysfunction has mainly been described in β2-microglobulin deficiency. Loss of β2-microglobulin disrupts receptor assembly and prevents stable surface expression [2]. In such patients, hypogammaglobulinemia and hypoalbuminemia reflect increased catabolism rather than renal or gastrointestinal protein loss. In contrast, pathogenic variants directly affecting the FcRn heavy chain encoded by *FCGRT* have not been clearly documented. Here, we report a child with a homozygous *FCGRT* mutation presenting with persistent hypogammaglobulinemia and hypoalbuminemia in the absence of protein loss, thereby providing genetic evidence of FcRn deficiency in humans.

A 7-year-old girl was evaluated for chronic hypoproteinemia, presenting with abdominal pain and anorexia. Her medical history was notable for allergic rhinitis and asthma, without recurrent or severe infections. Physical examination showed normal growth parameters (weight, 26.5 kg [− 0.7 SD]; height, 126 cm [− 0.6 SD]) and no evidence of edema, ascites, or organomegaly. Laboratory studies demonstrated markedly reduced serum albumin and IgG levels, with preserved IgA and IgM concentrations. Serum IgE was markedly elevated, accompanied by peripheral eosinophilia. Laboratory findings did not support secondary causes of hypoproteinemia. There was no proteinuria on urinalysis. Celiac serology was negative. Lipid profile, ophthalmologic examination, and abdominal ultrasonography were unremarkable, making malabsorption and disorders such as abetalipoproteinemia unlikely (Table 1).

**Table 1:** Laboratory findings at initial presentation

Parameters	Results
WBC (µl)	11,700
Hgb (g/dL)	12.1
Platelets (µl)	307,000
ALC/ANC (µl)	3000/7100
Eosinophils (µl)	600
Total protein/Albumin (g/L)	43.8/24.2
AST/ALT (U/L)	29/22
BUN/Creatinine (mg/dL)	6.8/0.39
IgA (g/L)	1.3
IgG (g/L)	1.2
IgM (g/L)	0.97
IgE (IU/mL)	5820
Anti-tTG IgA (U/mL)	0.56
Total cholesterol (mg/dL)	187
Triglyceride (mg/dL)	65
HDL/LDL (mg/dL)	61/113
Fecal alpha-1 antitrypsin (mg/g)	4
Fecal calprotectin (µg/g)	21

*WBC* white blood cell count, *Hgb* hemoglobin, *ALC* absolute lymphocyte count, *ANC* absolute neutrophil count, *AST* aspartate aminotransferase, *ALT* alanine aminotransferase, *Ig* Immunoglobulin, *anti-tTG* anti-tissue transglutaminase, *HDL* high-density lipoprotein, *LDL* low-density lipoprotein

Notably, fecal α1-antitrypsin and calprotectin levels were within the normal range, arguing against protein-losing enteropathy and active intestinal inflammation. Upper endoscopy and colonoscopy were macroscopically normal. Histologic evaluation revealed mild villous changes and focal eosinophilic infiltration without evidence of intestinal lymphangiectasia. Given the presence of abdominal pain and the possibility of food allergy–related gastrointestinal involvement, a stepwise elimination diet was initiated, resulting in improvement of gastrointestinal symptoms. However, serum albumin and IgG levels remained persistently low, supporting a non-enteric mechanism. Whole-exome sequencing identified a homozygous missense variant in *FCGRT*:c.661T > C, p.(Cys221Arg). To our knowledge, no biallelic variants in *FCGRT* have previously been reported in humans. During more than two years of follow-up, the patient did not develop severe or recurrent infections despite sustained IgG deficiency.

This clinical case demonstrates that *FCGRT* disruption mirrors the clinical and biochemical phenotype of FcRn deficiency while preserving IgA- and IgM-mediated immune function. It provides in vivo human evidence with translational relevance for understanding the long-term physiological and clinical consequences of FcRn-targeted therapies.

Sincerely,

## Data Availability

No datasets were generated or analysed during the current study.
